# Supplementary material for: LC–MS/MS Phytochemical Profiling, Antioxidant Activity, and Cytotoxicity of the Ethanolic Extract of Atriplex halimus L. against Breast Cancer Cell Lines: Computational Studies and Experimental Validation
Source: Pharmaceuticals (Basel). 2022 Sep 16;15(9):1156. doi: 10.3390/ph15091156 (PMC9503641; doi:10.3390/ph15091156)
Supplement: Supplementary file 1 [file pharmaceuticals-15-01156-s001.zip › Supplementary file S1.pdf]

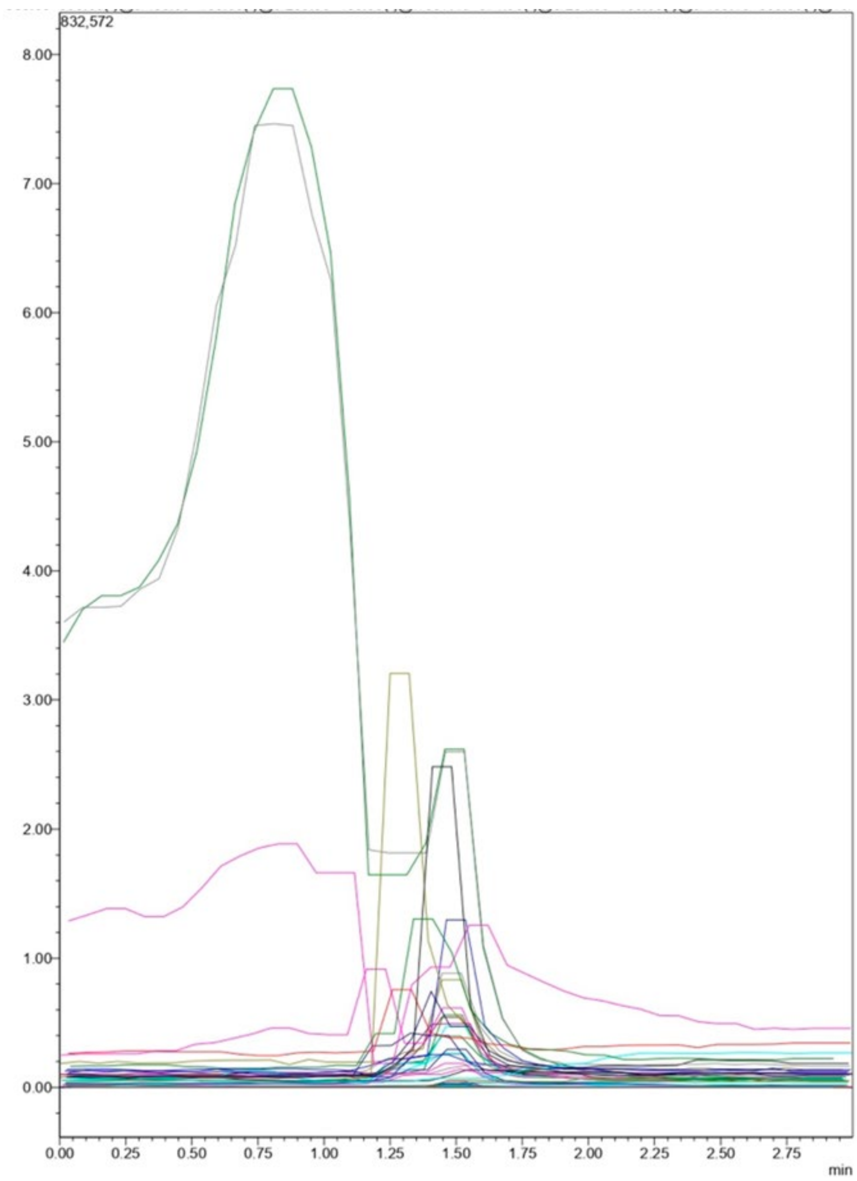

Figure S1. Selected fragments derive from the molecular weight of the polyphenol minus one (due to ESI- ionization) and their subsequent fragmentation in MS/MS experiment.

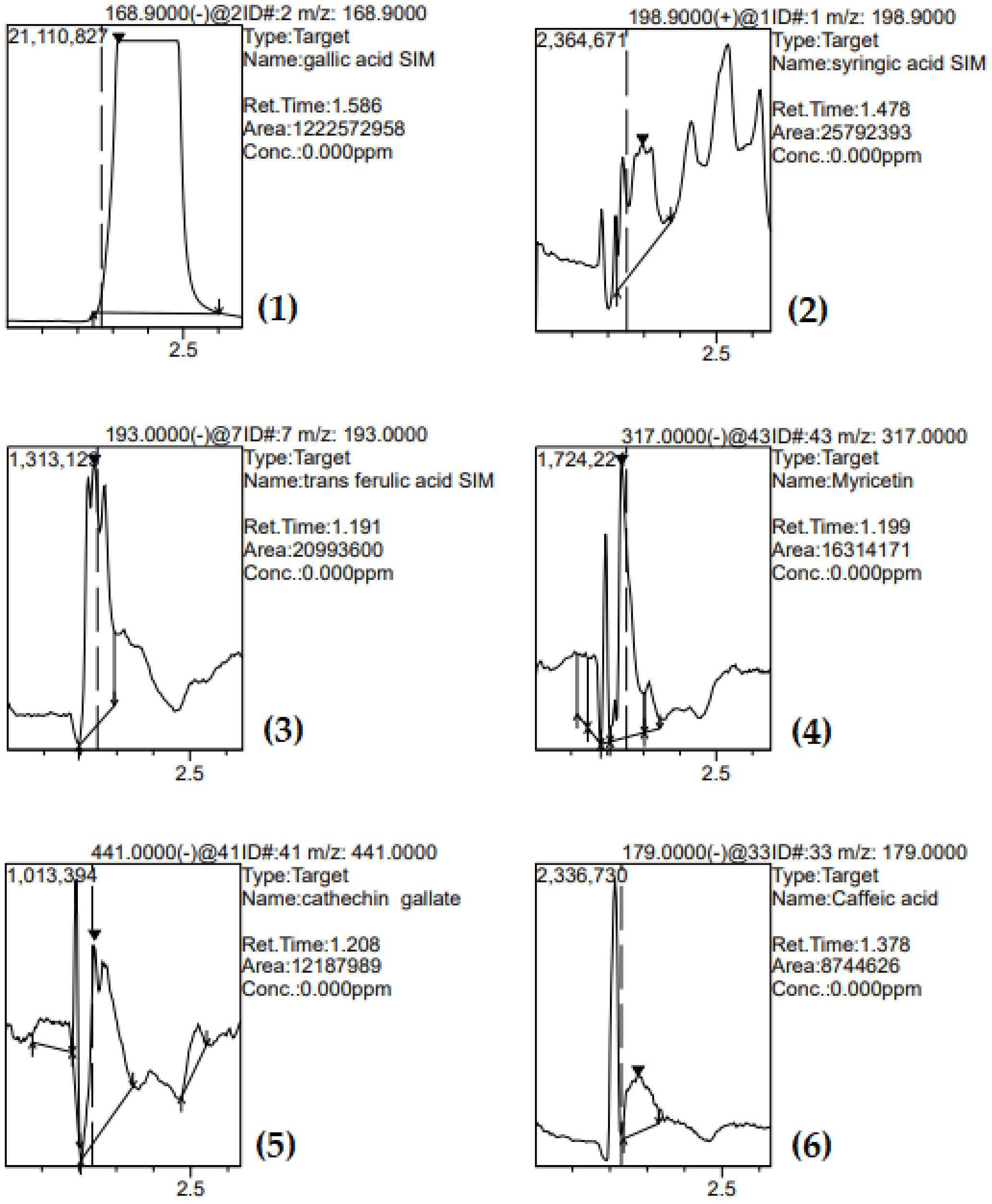

Figure S2. Screening of polyphenols from *A. halimus* ethanolic extract (AHEE) using LC-MS/MS. **(1)** Gallic acid, **(2)** Syringic acid, **(3)** *trans*-Ferulic acid, **(4)** Myricetin, **(5)** Catechin gallate, **(6)** Caffeic acid, **(7)** Chlorogenic acid, **(8)** Arbutin, **(9)** Trimethoxyflavone.

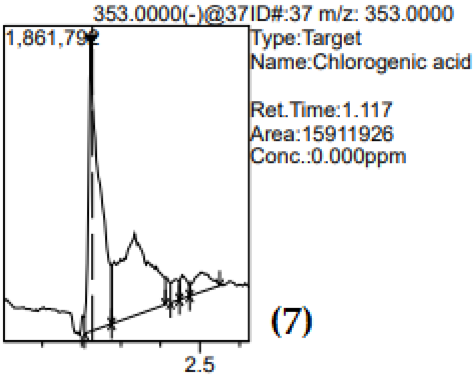

(7)

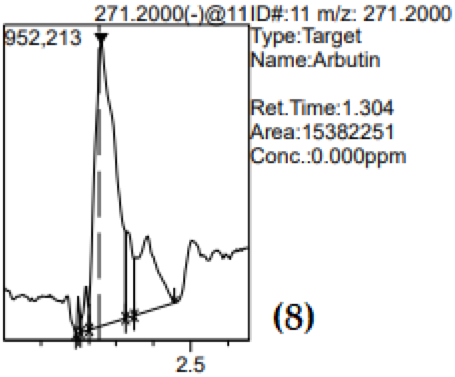

(8)

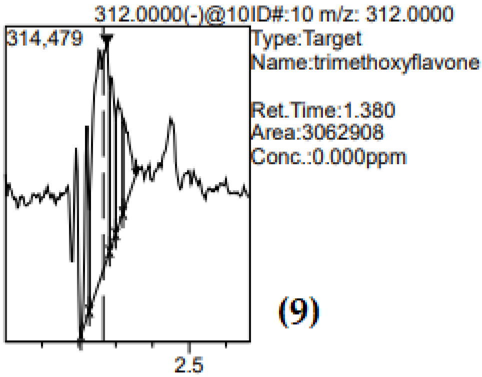

(9)

Figure S2. *Cont.*

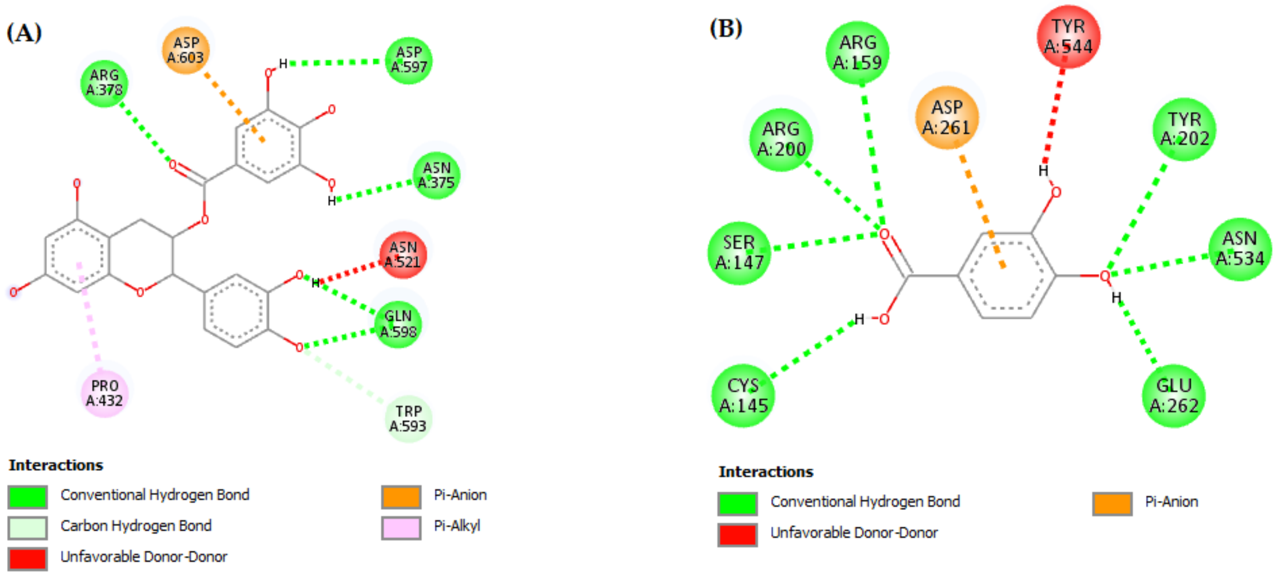

Figure S3. 2D binding interactions of the potent inhibitor from AHEE, Catechin gallate (**A**), and the native ligand, Protocatechuic acid (**B**), against Lipoxygenase protein **(PDB ID: 1N8Q)**

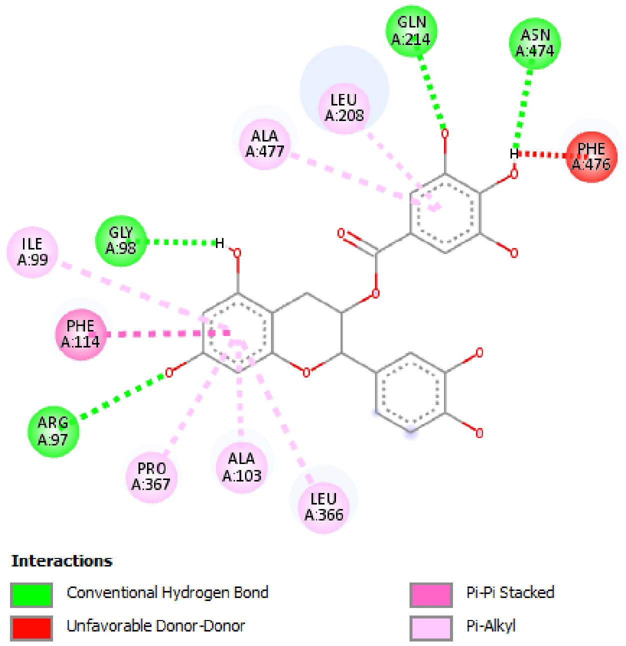

(A)

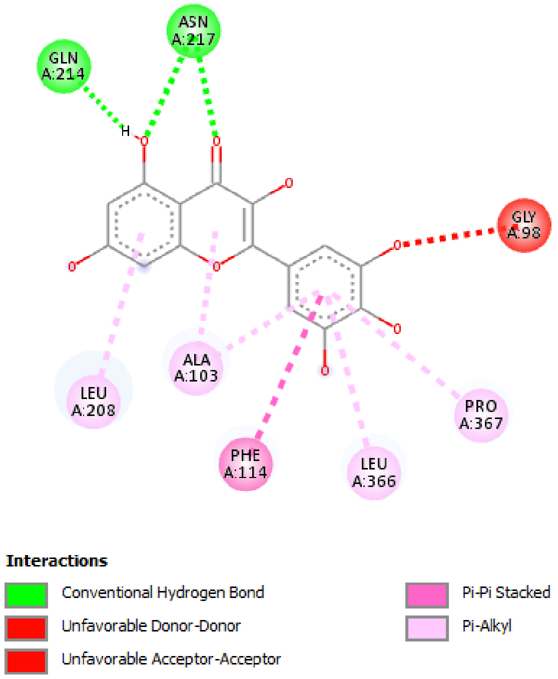

(B)

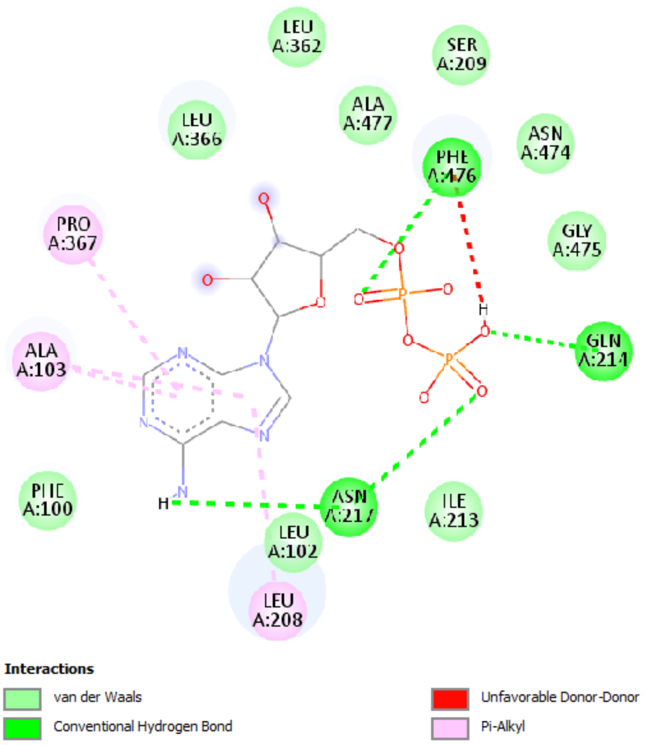

(C)

Figure S4. 2D binding interactions of the potent inhibitors from AHEE, Catechin gallate (**A**), Mericetin (**B**), and the native ligand, Warfarin (**C**), against CYP450 protein **(PDB ID: 1OG5)**

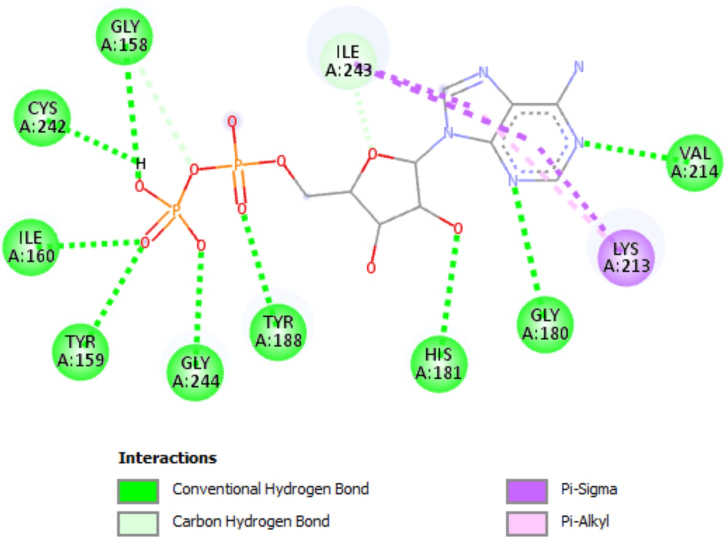

Figure S5. 2D binding interactions of Adenosine-5'-diphosphate (native ligand), against NADPH Oxidase protein **(PDB ID: 2CDU), * Our analysis revealed that none of the identified compounds in AHEE can inhibit the activity of NADPH Oxidase.**

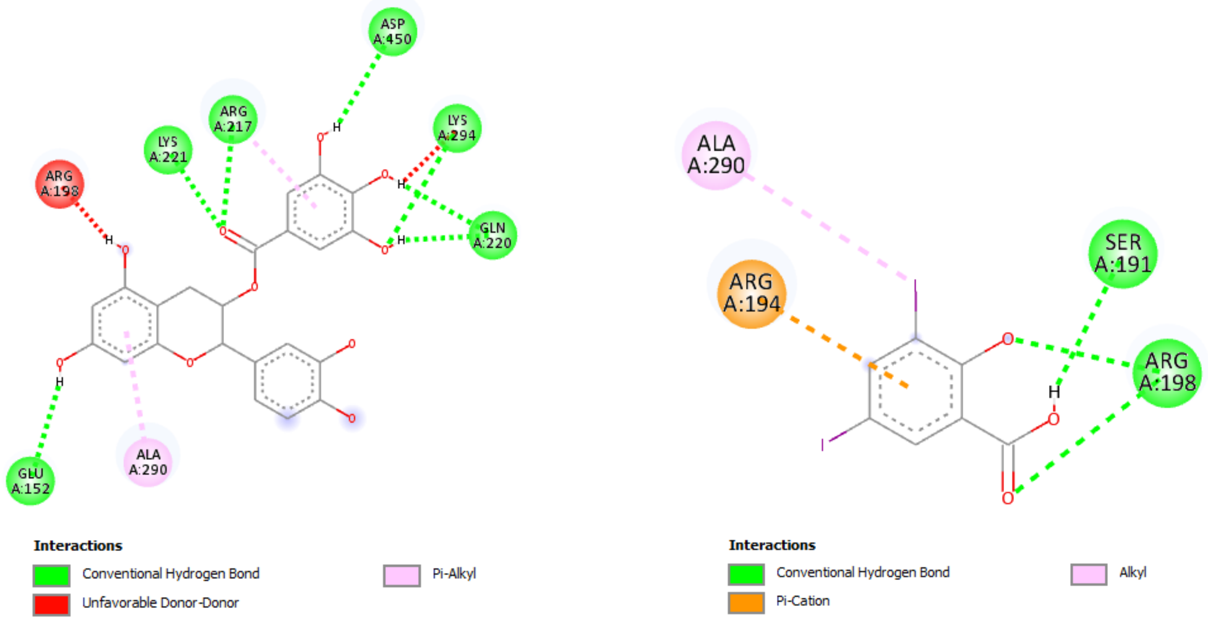

(A) **(B)**

Figure S6. 2D binding interactions of the potent inhibitor from AHEE, Catechin gallate (**A**), 3,5-Diiodosalicylic Acid (native ligand) (**B**), against Bovine Serum Albumin (BSA) protein **(PDB ID: 4JK4), * All the identified components were found to be potent inhibitors of BSA protein.**
