# Supplementary material for: LC–MS/MS Phytochemical Profiling, Antioxidant Activity, and Cytotoxicity of the Ethanolic Extract of Atriplex halimus L. against Breast Cancer Cell Lines: Computational Studies and Experimental Validation
Source: Pharmaceuticals (Basel). 2022 Sep 16;15(9):1156. doi: 10.3390/ph15091156 (PMC9503641; doi:10.3390/ph15091156)
Supplement: Supplementary file 1 [file pharmaceuticals-15-01156-s001.zip › Supplementary file S2 (LC-MS-MS Standards).pdf]

# = Shimadzu LabSolutions Quant. Browser Data Report =

Acquired by	: System Administrator
Data Acquired	: 09/02/2022 10:25:14
Sample Type	: Unknown
Sample Name	: blnk
Sample ID	:
Sample Amount	: 1
Dilution Factor	: 1
Vial#	: 100
Injection Volume	: 10 uL
Data Filename	: blnk_001.lcd
Method Filename	: polifenoli screening SIM C18.lcm
Processed by	: System Administrator
Modified Date	: 09/02/2022 10:30:16

198.9000(+)@1ID#:1 m/z: 198.9000

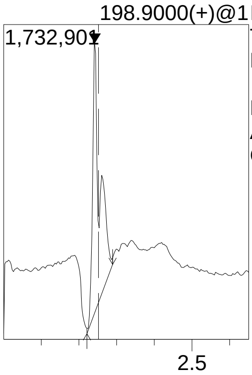

Type:Target
Name:syringic acid SIM

Ret.Time:1.210
Area:9965864
Conc.:0.000ppm

168.9000(-)@2ID#:2 m/z: 168.9000

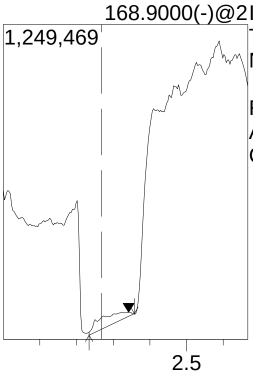

Type:Target
Name:gallic acid SIM

Ret.Time:1.710
Area:1130144
Conc.:0.000ppm

301.0000(-)@3ID#:3 m/z: 301.0000

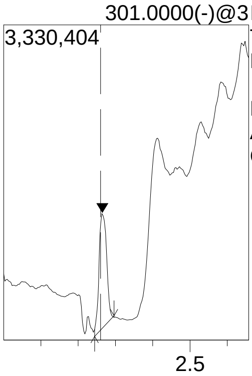

Type:Target
Name:quercetin sim

Ret.Time:1.324
Area:8656108
Conc.:0.000ppm

162.9000(-)@4ID#:4 m/z: 162.9000

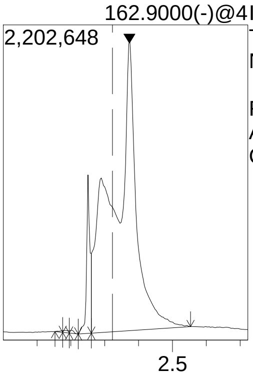

Type:Target
Name:p coumarci acid SIM

Ret.Time:1.865
Area:48649908
Conc.:0.000ppm

303.2000(-)@5ID#:5 m/z: 303.2000

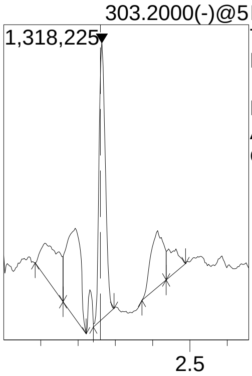

Type:Target
Name:oleochantal SIM

Ret.Time:1.318
Area:6695780
Conc.:0.000ppm

153.0500(-)@6ID#:6 m/z: 153.0500

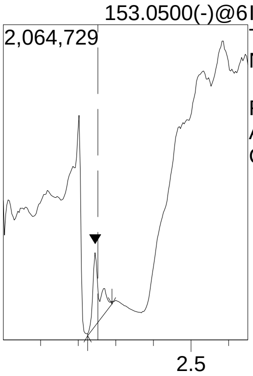

Type:Target
Name:hydroxytyrosol SIM

Ret.Time:1.225
Area:3104120
Conc.:0.000ppm

193.0000(-)@7ID#:7 m/z: 193.0000

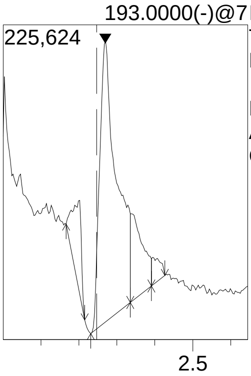

Type:Target
Name:trans ferulic acid SIM

Ret.Time:1.347
Area:3132694
Conc.:0.000ppm

539.0000(-)@8ID#:8 m/z: 539.0000

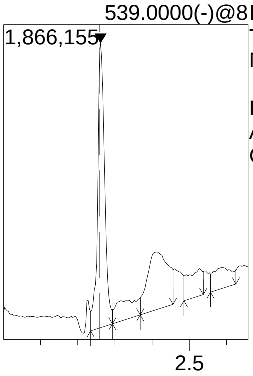

Type:Target
Name:oleuropein SIM

Ret.Time:1.307
Area:11035005
Conc.:0.000ppm

301.3000(-)@9ID#:9 m/z: 301.3000

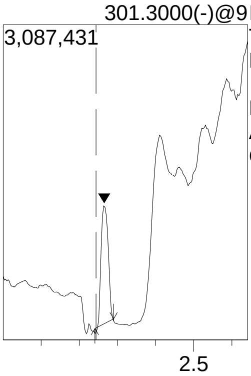

Type:Target
Name:Hesperetin

Ret.Time:1.327
Area:7417052
Conc.:0.000ppm

312.0000(-)@10ID#:10 m/z: 312.0000

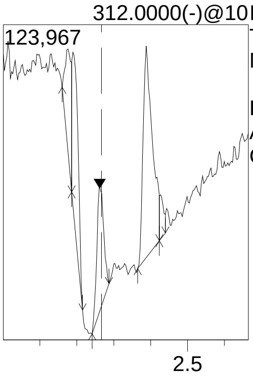

Type:Target
Name:trimethoxyflavone

Ret.Time:1.310
Area:315321
Conc.:0.000ppm

271.2000(-)@11ID#:11 m/z: 271.2000

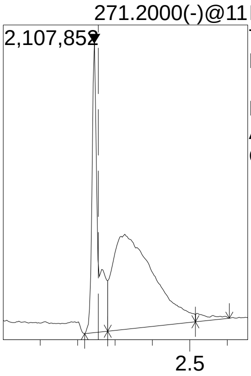

Type:Target
Name:Arbutin

Ret.Time:1.222
Area:10880437
Conc.:0.000ppm

359.0000(-)@12ID#:12 m/z: 359.0000

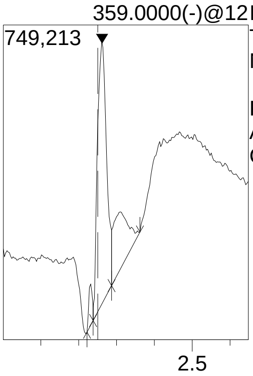

Type:Target
Name:Rosmarinic acid

Ret.Time:1.309
Area:5241208
Conc.:0.000ppm

455.0000(-)@13ID#:13 m/z: 455.0000

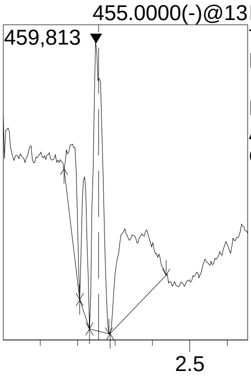

Type:Target
Name:ursolic acid

Ret.Time:1.245
Area:2545876
Conc.:0.000ppm

269.0000(-)@14ID#:14 m/z: 269.0000

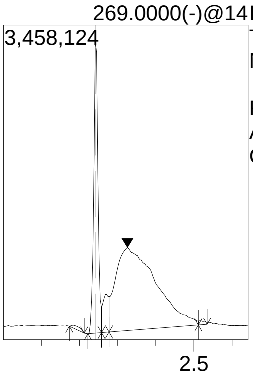

Type:Target
Name:Apigenin

Ret.Time:1.629
Area:34180811
Conc.:0.000ppm

537.1000(-)@15ID#:15 m/z: 537.1000

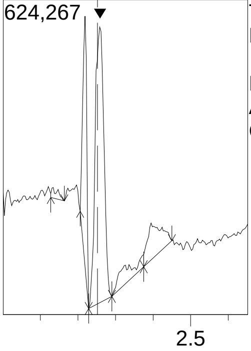

Type:Target
Name:Amentoflavone

Ret.Time:1.294
Area:3885853
Conc.:0.000ppm

284.9000(-)@16ID#:16 m/z: 284.9000

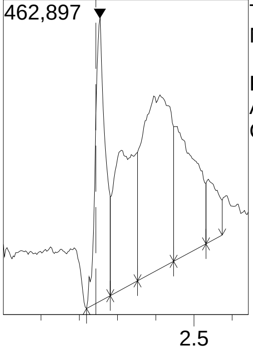

Type:Target
Name:luteoilin

Ret.Time:1.269
Area:3756504
Conc.:0.000ppm

463.1000(-)@17ID#:17 m/z: 463.1000

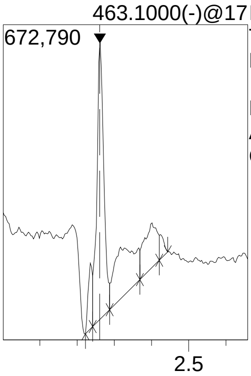

Type:Target
Name:quercetin-3-O-glucoside

Ret.Time:1.306
Area:3785982
Conc.:0.000ppm

477.0000(-)@18ID#:18 m/z: 477.0000

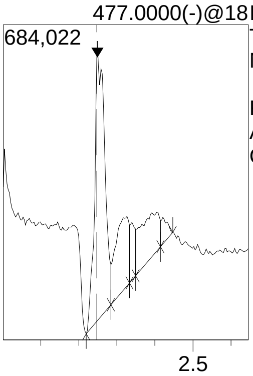

Type:Target
Name:quercetin-3-O- glucuronic acid

Ret.Time:1.245
Area:5445222
Conc.:0.000ppm

609.1000(-)@19ID#:19 m/z: 609.1000

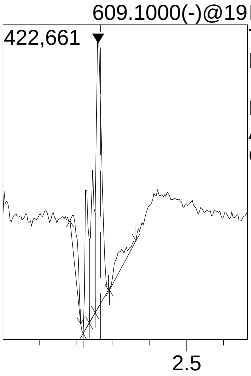

Type:Target
Name:Kaempferol-3-O-glucose

Ret.Time:1.299
Area:1835825
Conc.:0.000ppm

609.1000(-)@20ID#:20 m/z: 609.1000

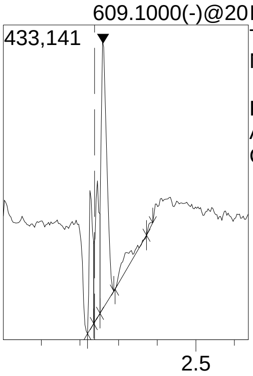

Type:Target
Name:Quercetin-3-O.hexose deoxyhex

Ret.Time:1.299
Area:1872083
Conc.:0.000ppm

623.1000(-)@21ID#:21 m/z: 623.1000

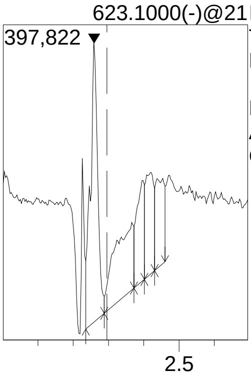

Type:Target
Name:Isorhamnetin- 3-O Rutinoside

Ret.Time:1.296
Area:2492977
Conc.:0.000ppm

447.1000(-)@22ID#:22 m/z: 447.1000

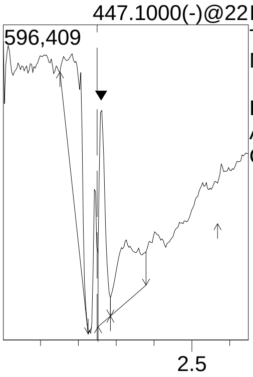

Type:Target
Name:Isorhamnetin-7-O- Pentose

Ret.Time:1.302
Area:2032227
Conc.:0.000ppm

447.1000(-)@23 ID#:23 m/z: 447.1000

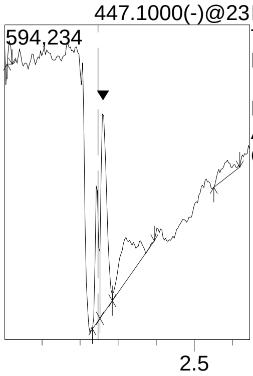

Type:Target
Name:luteoilin 7-O-glucoside

Ret.Time:1.301
Area:1809045
Conc.:0.000ppm

461.1000(-)@24 ID#:24 m/z: 461.1000

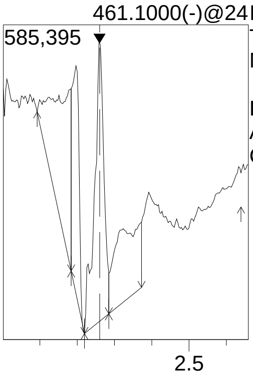

Type:Target
Name:Kaempferol-3-O-glucuronic acid

Ret.Time:1.300
Area:4478686
Conc.:0.000ppm

417.1000(-)@25 ID#:25 m/z: 417.1000

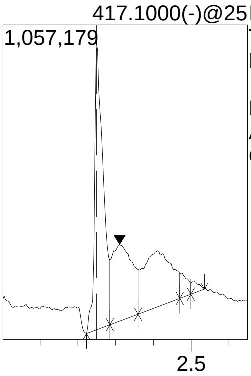

Type:Target
Name:Kaempferol-3-O-pentose

Ret.Time:1.553
Area:4890342
Conc.:0.000ppm

593.1000(-)@26 ID#:26 m/z: 593.1000

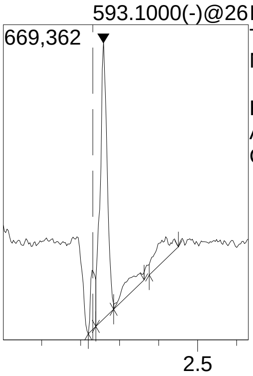

Type:Target
Name:Kaempferol-3-O-hexose deohyhe

Ret.Time:1.293
Area:3620339
Conc.:0.000ppm

153.4000(-)@27 ID#:27 m/z: 153.4000

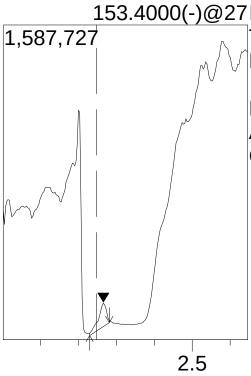

Type:Target
Name:Tyrosol

Ret.Time:1.330
Area:881490
Conc.:0.000ppm

153.0000(-)@28 ID#:28 m/z: 153.0000

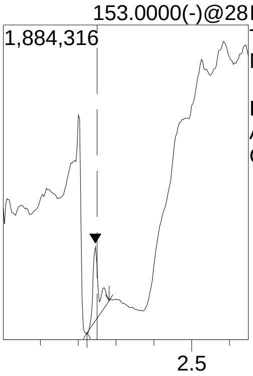

Type:Target
Name:Protocathecoic acid

Ret.Time:1.226
Area:2442478
Conc.:0.000ppm

167.0000(-)@29 ID#:29 m/z: 167.0000

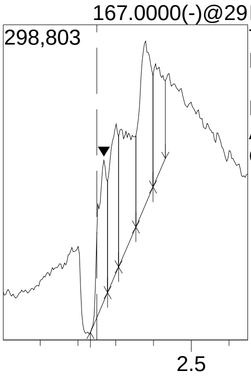

Type:Target
Name:Vanillic acid

Ret.Time:1.344
Area:1056318
Conc.:0.000ppm

197.0000(-)@30 ID#:30 m/z: 197.0000

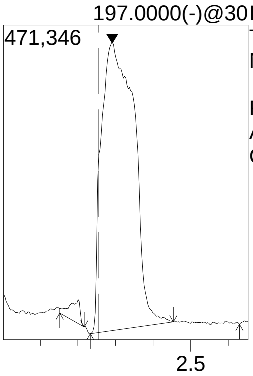

Type:Target
Name:syringic acid

Ret.Time:1.457
Area:13214001
Conc.:0.000ppm

137.0000(-)@31ID#:31 m/z: 137.0000

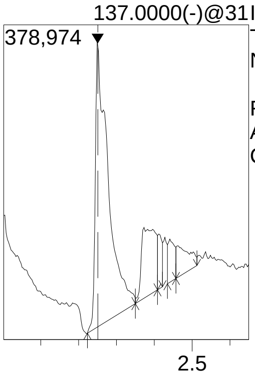

Type:Target
Name:p-hydroxybenzoic\salicilic acid

Ret.Time:1.253
Area:4099095
Conc.:0.000ppm

153.0000(-)@32ID#:32 m/z: 153.0000

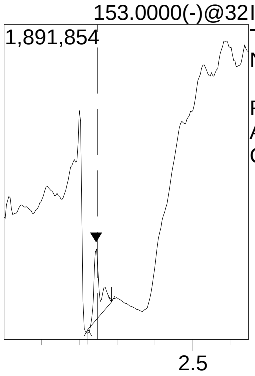

Type:Target
Name:Gentisic acid

Ret.Time:1.224
Area:2466888
Conc.:0.000ppm

179.0000(-)@33ID#:33 m/z: 179.0000

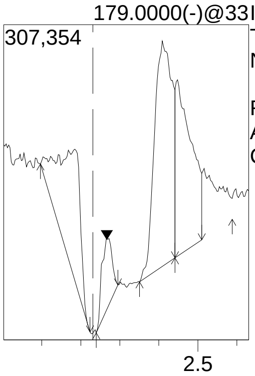

Type:Target
Name:Caffeic acid

Ret.Time:1.334
Area:624763
Conc.:0.000ppm

223.0000(-)@34ID#:34 m/z: 223.0000

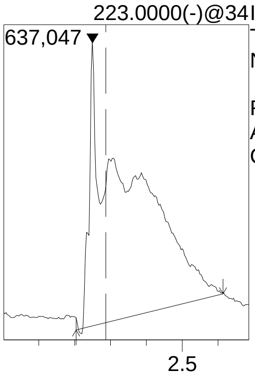

Type:Target
Name:Sinapic acid

Ret.Time:1.248
Area:22474079
Conc.:0.000ppm

193.0000(-)@35ID#:35 m/z: 193.0000

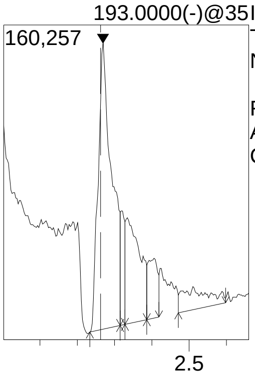

Type:Target
Name:Ferulic acid

Ret.Time:1.344
Area:1858114
Conc.:0.000ppm

147.0000(-)@36ID#:36 m/z: 147.0000

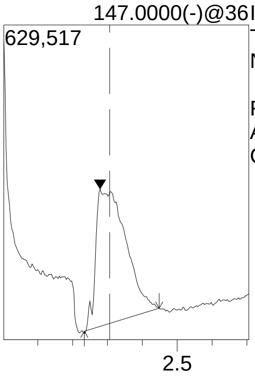

Type:Target
Name:Trans-cinnamic acid

Ret.Time:1.394
Area:8150911
Conc.:0.000ppm

353.0000(-)@37ID#:37 m/z: 353.0000

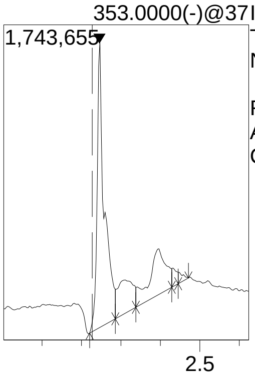

Type:Target
Name:Chlorogenic acid

Ret.Time:1.228
Area:10100505
Conc.:0.000ppm

289.0000(-)@38ID#:38 m/z: 289.0000

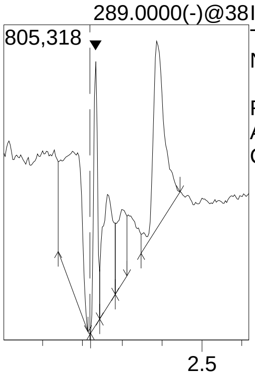

Type:Target
Name:cathechin\epicathechin

Ret.Time:1.165
Area:2244624
Conc.:0.000ppm

457.0000(-)@39 ID#:39 m/z: 457.0000

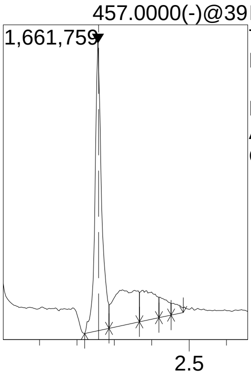

Type:Target
Name:gallocathechin\epigallocathechin

Ret.Time:1.281
Area:9384095
Conc.:0.000ppm

305.0000(-)@40 ID#:40 m/z: 305.0000

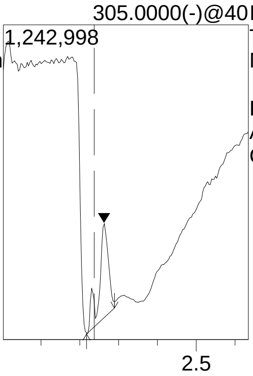

Type:Target
Name:gallocathechin\epigallocathechin

Ret.Time:1.312
Area:3149679
Conc.:0.000ppm

441.0000(-)@41 ID#:41 m/z: 441.0000

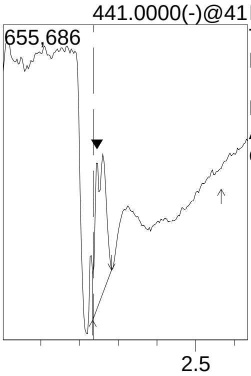

Type:Target
Name:cathechin gallate

Ret.Time:1.226
Area:2775063
Conc.:0.000ppm

577.0000(-)@42 ID#:42 m/z: 577.0000

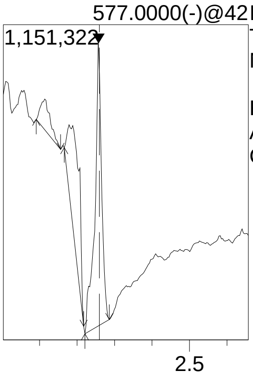

Type:Target
Name:Procianidin

Ret.Time:1.286
Area:6329157
Conc.:0.000ppm

317.0000(-)@43 ID#:43 m/z: 317.0000

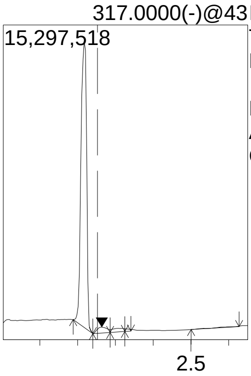

Type:Target
Name:Myricetin

Ret.Time:1.320
Area:2663290
Conc.:0.000ppm

285.0000(-)@44 ID#:44 m/z: 285.0000

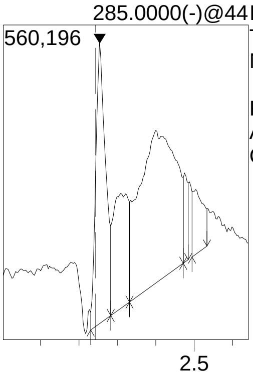

Type:Target
Name:Kaempeferol

Ret.Time:1.271
Area:4372828
Conc.:0.000ppm

609.0000(-)@45 ID#:45 m/z: 609.0000

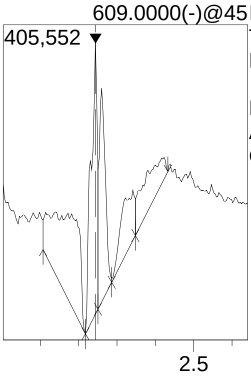

Type:Target
Name:rutin

Ret.Time:1.219
Area:1919634
Conc.:0.000ppm

579.0000(-)@46 ID#:46 m/z: 579.0000

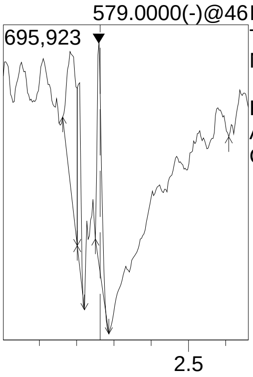

Type:Target
Name:narigin

Ret.Time:1.296
Area:1676944
Conc.:0.000ppm
